# Strategies and Methodologies for Developing Microbial Detoxification Systems to Mitigate Mycotoxins

**DOI:** 10.3390/toxins9040130

**Published:** 2017-04-07

**Authors:** Yan Zhu, Yousef I. Hassan, Dion Lepp, Suqin Shao, Ting Zhou

**Affiliations:** Guelph Research and Development Centre, Agriculture and Agri-Food Canada, Guelph, ON N1G5C9, Canada; yan.zhu@agr.gc.ca (Y.Z.); yousef.hassan@agr.gc.ca (Y.I.H.); dion.lepp@agr.gc.ca (D.L.); Suqin.Shao@agr.gc.ca (S.S.)

**Keywords:** mycotoxin, detoxification, biodegradation, biotransformation, enzyme, microorganism identification

## Abstract

Mycotoxins, the secondary metabolites of mycotoxigenic fungi, have been found in almost all agricultural commodities worldwide, causing enormous economic losses in livestock production and severe human health problems. Compared to traditional physical adsorption and chemical reactions, interest in biological detoxification methods that are environmentally sound, safe and highly efficient has seen a significant increase in recent years. However, researchers in this field have been facing tremendous unexpected challenges and are eager to find solutions. This review summarizes and assesses the research strategies and methodologies in each phase of the development of microbiological solutions for mycotoxin mitigation. These include screening of functional microbial consortia from natural samples, isolation and identification of single colonies with biotransformation activity, investigation of the physiological characteristics of isolated strains, identification and assessment of the toxicities of biotransformation products, purification of functional enzymes and the application of mycotoxin decontamination to feed/food production. A full understanding and appropriate application of this tool box should be helpful towards the development of novel microbiological solutions on mycotoxin detoxification.

## 1. Introduction

Over the past several decades, research interest in the mitigation of mycotoxins, the toxic secondary metabolites produced by specific fungi, has continuously increased due to concerns over human and animal health, economic losses and food safety and security [1]. Important mycotoxins include aflatoxin B_1_ (AFB_1_), aflatoxin G_1_ (AFG_1_), ochratoxin A (OTA), deoxynivalenol (DON), nivalenol (NIV), fumonisin (FUM), zearalenone (ZEA), patulin (PAT) and citrinin (CIT), which are mainly produced by the fungal genera *Aspergillus*, *Fusarium* and *Penicillium*. It has been estimated that the economic costs of crop losses from major mycotoxins (aflatoxins, fumonisins and deoxynivalenol) in the United States are as great as $932 million per year, in addition to mitigation costs of $466 million and livestock costs of $6 million [2]. In Europe, although no data regarding economic losses caused by mycotoxins are available, the direct and indirect losses due to a wheat epidemic in 1998 in Hungary were estimated at 100 million euros [3]. Moreover, severe human health effects can result from the exposure of mycotoxins through either ingestion, absorption or inhalation routes [4]. In 2015, the Rapid Alert System for Food and Feed (RASFF) reported 475 notifications in Europe on mycotoxin exposure in food, most related to the presence of aflatoxins [5].

Pre-harvest control of mycotoxin production and post-harvest mitigation of contamination are the main strategies to limit mycotoxins in food and feed. Good agricultural practices (GAPs), including crop rotation, soil management, choice of varieties and correct fungicide use, have been recommended by the European Commission (EC) to prevent the contamination of *Fusarium* toxins in cereals [6]. Strategies for post-harvest mitigation can be categorized as chemical, physical and biological. Chemical strategies use acids, bases, oxidizing agents, aldehydes or bisulfite gases to change the structure of mycotoxins, which has led to increased public concerns over the chemical residues in food and feed. Furthermore, negative effects on the nutrition and palatability of food and feed may result from chemical treatments [7,8,9]. For physical strategies, the application of adsorption agents has become popular since the European Union (EU) allowed substrates that suppress or reduce the absorption, promote the excretion of mycotoxins or modify their mode of action to be used as feed additives [10]. However, the efficacy of adsorption agents in reducing mycotoxin contamination is variable, and most of the commercial binding agents presented no sufficient effect against DON [11]. Compared with chemical and physical approaches, biological detoxification methods, which biotransform mycotoxins into less toxic metabolites, are generally more specific, efficient and environmentally friendly.

As more and more researchers enter the field of biodetoxification, guidance is needed on the various approaches and methodologies that may be employed. In this paper, we aim to review the entire process of the discovery and development of biological mitigation systems, focusing on the strategies and methodologies at each research stage from initial microorganism screening to final application. Although the methods in the steps of enrichment, isolation, strain identification, chemical analysis, mycotoxin biotransformation, toxicity evaluation and enzyme extraction were developed based on specific mycotoxins and detoxifying microorganisms, the approaches used should be generally applied to studies seeking microbial-based solutions for mycotoxin mitigation.

## 2. Workflow

**Figure 1:**
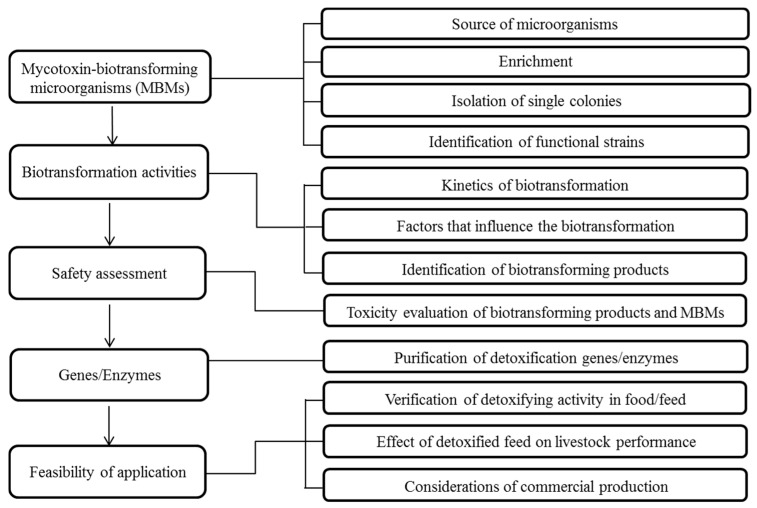
Workflow of research on microbial detoxifications of mycotoxins.

An integrated research program on the microbial detoxification of mycotoxins is expected to proceed through up to five stages. First, mycotoxin-biotransforming microorganisms (MBMs) or consortia should be obtained and identified from either environmental sources or be screened from a group of previously-identified candidates. Secondly, the efficacy and influencing factors on the detoxification activities of the selected microorganisms, as well as the biotransformation products should be investigated and identified. In the third stage, safety assessments of both functional strains and biotransformation products should be performed. Biotransformation may not necessarily result in a less toxic secondary product, so the reduced toxicity of the biotransformation products must also be confirmed. In the fourth, and potentially most difficult, stage, the enzymes responsible for the biotransformation are isolated, identified and/or cloned and expressed. Finally, the feasibility of mycotoxin-detoxifying applications in food and feed should be validated. The complete workflow is outlined in Figure 1. It should be noted that it is not necessary to endure all five stages in an individual research project. For example, studies on the detoxification activities of known strains would not require the first screening stage. Furthermore, the isolation of the enzymes may be optional if the functional microorganisms are to be used in the application.

## 3. Strategies and Methodologies

### 3.1. Sources of Microorganisms

Undoubtedly, the successful isolation of MBMs is one of the critical steps of the whole research project. A careful selection of environmental sources more likely to harbor MBMs should increase the probability of finding functional microorganisms. Previous studies have isolated microorganisms from a range of environmental [12,13,14,15,16,17,18,19,20,21,22,23,24,25,26,27,28,29], plant [30,31,32,33,34,35,36,37,38] and animal [39,40,41,42,43,44,45,46,47,48] sources.

The existence of MBMs was initially hypothesized based on the fact that chemically-stable mycotoxins do not accumulate in agricultural soil [25,30,46]. Soil is therefore a likely reservoir for functional MBMs, in particular that on which crops susceptible to mycotoxigenic fungi have been grown previously. In addition, nearby aqueous environments to agricultural soil have also been used for the successful isolation of DON-degrading bacteria [15].

Rumen fluid and intestinal contents are hosts to a diverse microbiota, which not only contribute to host metabolism, but have also been shown in some cases to have biotransformation activities. This is partly demonstrated by the reduced sensitivity of ruminants to mycotoxins and in particular to trichothecenes [40,41]. Some early studies reported that the rumen microbiota has the capability to transform a wide range of mycotoxins, including OTA, ZEA, DON, T-2 toxin and diacetoxyscirpenol [40,42]. In addition, chicken large intestinal contents have been shown to harbor functional bacteria with DON deepoxidation activity [39,43].

From a practical standpoint, it is advantageous to isolate MBMs from an environment similar to that to which they will be applied, as they will then be more likely to grow and express detoxification activities during application. For example, *Bacillus subtilis* strain UTBSP1, isolated from mature pistachio nut fruits in Iran, showed AFB_1_ biotransformation activity in this agriculture crop [38]. Similarly, the patulin in apple juice was degraded into less toxic *E*- and *Z*-ascladiol by *Gluconobacter oxydans*, a bacterium isolated from rotten apple puree [37].

In contrast to screening microbes in a particular environment, an alternative strategy is to focus on only a certain taxonomic group [19,35,45,49] or already available strains [50,51,52,53]. These strains may possess specific properties to facilitate isolation or provide additional benefits to commercial applications. For instance, *Bacillus* spp. have been targeted due to their tolerance of adverse environmental conditions, application as probiotics, antimicrobial activities and the production of insect toxins and extracellular enzymes [19,35,45,47]. Petchkongkaew et al. (2008) [35] screened for *Bacillus* spp. from Thai fermented soybean using Gram-staining and the API 50CH system, and they isolated three strains with highly efficient AFB_1_ and OTA biotransformation capabilities. Lactic acid bacteria (LAB) are also good candidates for mycotoxin biotransforming bacteria since their additional probiotic properties promote their application in food and feed. *Pediococcus parvulus* UTAD 473 was selected from a collection of 19 LAB strains and has the ability to biotransform 90% of OTA to the less toxic OTα [53]. An isolated CIT biotransforming bacterium, *Moraxella* sp. MB1, has the particular benefit of being able to perform detoxification in solvent environment, as it belongs to the class of organic solvent-tolerant microorganisms (OSTMs) [50]. Rodriguez et al. (2011) [51] considered strains belonging to *Rhodococcus*, *Pseudomonas* and *Brevibacterium*, which have the capability to biotransform aromatic compounds, to have a higher potential to degrade mycotoxins. The authors successfully obtained *Brevibacterium casei* RM101 and *Brevibacterium linens* DSM 20425^T^, which can completely biotransform OTA at concentrations up to 40 µg/mL [51].

### 3.2. Enrichment

Due to the complexity of microbial communities obtained from environmental or animal sources, screening may fall into a “trial-and-error” phase, leading to high costs of labour and consumables. Enrichment of particular microorganism taxa, by either enhancing the growth of potentially functional strains or supressing unwanted ones, is therefore used to reduce the diversity of the microbial consortium.

Enrichment strategies vary based on sources of microbiota, the mycotoxins to be targeted and possible biotransformation pathways (Table 1). Media used for enrichment may be classified into three categories: nutrient medium, minimal medium and sole carbon source medium. The latter normally contains the salt medium with or without vitamin mixtures as the medium base and supplemented with the specific mycotoxin or related compounds as a sole carbon source.

The application of selective pressures encountered in the environments of plant materials contaminated with mycotoxigenic fungi is an effective strategy to screen for functional microbes. In this way, the potential functional strains may become predominant, which aids in further isolation. He et al. (2016) [12] hypothesized that DON-biotransforming microorganisms would have the ability to tolerate and grow in the presence of DON-producing *Fusarium graminearum* and using this approach eventually isolated *Devosia mutans* 17-2-E-8, a new bacterial species that detoxifies DON into 3-*epi*-DON with high efficiency. An in situ plant enrichment (isPE) strategy has been developed to screen functional strains on wheat heads that were artificially contaminated with DON and grown in situ for one month. This approach yielded 17 colonies with DON-biotransformation activities among 60 colonies examined [32]. In order to screen for biotransforming bacteria in the chicken intestinal tract, in vivo enrichment was performed by feeding the chickens moldy wheat contaminated with DON (10 mg/kg), which was shown to improve the activities of the digesta contents in biotransforming DON [43].

Another well-known and effective enrichment strategy that has been widely employed involves the use of a specific mycotoxin as a sole carbon source in a basal mineral medium. It has been reported that DON, ZEA, PAT and CIT can be utilized as a sole carbon source and ultimately biotransformed by a number of microbial strains [13,15,18,23,24,26,27,29,32,41,54]. Due to the high cost and toxicity of many pure mycotoxins, chemically-related compounds may be substituted as the sole carbon source when screening for potential MBMs [25,46,55,56]. One successful application of this strategy involved the use of coumarin as a surrogate for AFB_1_, which shares the basic structure of this extremely toxic mycotoxin, but is a much safer and less expensive alternative [25,46]. Guan et al. (2008) [46] first developed this strategy to isolate 25 single colonies grown in medium supplemented with coumarin as the sole carbon source, which were all shown to have AFB_1_ biotransformation activities (between 9.18% and 82.50%), as determined by HPLC, indicating the highly selective and accurate nature of this method.

Individual or combinations of antibiotics can also be used as a selective factor to inhibit the growth of unwanted microorganisms according to the different antimicrobial spectra [14,28,43]. Yu et al. (2010) [43] investigated the growth and DON-biotransformation activity of a bacterial culture treated with different combinations of 10 antibiotics, at various concentrations, demonstrating that one combination of virginiamycin (20 µg/mL), lincomycin (60 µg/mL) and salinomycin (5 µg/mL) significantly reduced microbial growth, without a loss of biotransforming activity.

Heat treatment is another effective method to enrich for heat-resistant strains, such as *Bacillus* spp. [14,19]. In order to enrich for desirable *Bacillus* strains with mycotoxin biotransforming activities, samples were treated at 80 °C for 15 min to inactive non-thermophiles [19,45].

The changes that occur in a microbial community following enrichment, such as the anticipated dominance of functional microbes and the decrease in the population diversity, are often difficult to track using traditional microbiological approaches. Fortunately, a number of molecular techniques, such as polymerase chain reaction with denaturing gradient gel electrophoresis (PCR-DGGE), terminal restriction fragment length polymorphism (T-RFLP) or 16S rRNA gene sequencing, can be applied to assess the microbial diversity and guide the screening process [14,17,43]. By monitoring the number of bands in PCR-DGGE bacterial profiles, Yu et al. (2010) [43] were able to show a reduction in the diversity of a microbial community with DON-biotransformation activity following several rounds of antibiotics and medium-based selection. Guided by PCR-DGGE, 10 positive isolates were finally obtained from 196 single colonies, which is more efficient than traditional blind screenings.

### 3.3. Isolation of Single Colonies

Once an enriched microflora containing MBMs is obtained, further screening to isolate single active colonies can be performed. Traditional plating methods have been widely used for this purpose. In order to selectively support the growth of potential MBMs, mycotoxins or mycotoxin-like compounds have also been introduced as sole carbon sources [26,27,31,34,46,54,58,62] or used to apply selective pressure in media containing another carbon source [47,60], as mentioned above. A summary of the various media, isolation strategies and isolated functional strains is given in Table 1.

Due to the physiological characteristics of the potential MBMs, they may be difficult to culture under the conditions used in the laboratory [63]. Efforts such as extending the incubation time, choosing media with inorganic nitrogen sources and changing the solidifying agent in the media, have been adopted to improve culturability [13,14,32,57]. The use of longer incubation times (i.e., >5 days) could help in the isolation of slow-growing bacteria, which are prone to suppression by more predominant bacteria [13,14]. In regard to the media-solidifying agent, it was reported that gellan gum, rather than agar, better supported the growth of soil-based bacteria [64]. Ito et al. (2012) [32] isolated a DON-biotransforming strain, *Marmoricola* sp. MIM116, from 1/3 R2A-gellan gum media and also observed better growth of the strain in media containing gellan gum as the solidification agent as compared with agar.

The mycotoxin biotransformation activities of isolated microbes are typically detected by chemical analysis methods, such as TLC, HPLC and LC-MS, which will be discussed further below. Some rapid detection methods have also been developed [34,50,62]. The basic concepts for these methods were originally based on the Kirby–Bauer disc plate diffusion assay. Devi et al. (2006) [50] examined six marine bacteria belonging to *Moraxella* spp. using the disc plate diffusion assay with 50 µg/disk of CIT. A strain showing high tolerance to mycotoxin was selected and examined for biotransformation activity. In another study screening for MBMs, a sterile filter paper disk inoculated with a culture was incubated on a nutrient agar plate coated with 0.4 mL of AFB_1_ (10 µg/mL). Another successful application involved visualizing single colonies able to biotransform AFB_1_ through the disappearance in fluorescence surrounding the colony, which is contributed by the coumarin ring structure in AFB_1_, indicating the degradation of AFB_1_ [34]. Similarly, this method has been applied to screening single strains with OTA biotransformation activity, which indicated that none of the strains had the ability to break the isocoumarin ring of OTA [62].

### 3.4. Identification of Functional Microorganisms

Microbial isolates capable of biotransforming mycotoxins may be identified and further characterized based on genome sequence information, which can range from the analysis of a single marker gene to entire genomes. The most commonly-used marker gene for bacterial identification and phylogenetic analysis is the 16S small ribosomal RNA subunit gene (rDNA) [65], which can be used to characterize both individual isolates and complex microbiota, though other conserved genes, such as *rpoA*, may also be used [66]. Several characteristics make the 16S gene well suited: it is present among all bacteria; it contains regions of a conserved sequence that allow for the design of universal PCR primers, but is also interspersed with regions of variability sufficient to discern phylogenetic relationships often to the genus and, occasionally, the species, level [67,68,69]. Furthermore, several databases of high-quality full-length 16S rDNA sequences and taxonomical assignments have been developed, such as SILVA [70], the Ribosomal Database Project (RDP) [71] and GreenGenes [72], and many bioinformatics tools are available for processing the sequence data. Many universal 16S primers are available in the literature, and several systematic comparisons have been carried out to evaluate their performance in terms of taxonomic coverage and discriminatory power [73,74,75].

The general workflow for taxonomic classification of a single isolate involves amplification of the 16S gene region, cloning of the amplicon and subsequent sequencing by the Sanger method. The taxonomy is then assigned by comparison of the sequence against one of the established reference databases (e.g., SILVA, RDP or GreenGenes), in which the taxonomy has already been established.

Next-generation DNA sequencing (NGS) technologies now allow for the taxonomic classification of complex microbiota by sequencing of the rDNA gene en masse, with the added benefit that the rDNA amplicons can be sequenced directly without the need for a cloning step. This approach may be useful during different stages of the isolation process to examine mixed cultures capable of biotransformation and to identify candidate bacteria that might possess activity. Illumina technology is currently the highest throughput and most cost-effective method for microbiota sequencing and can sequence rDNA amplicons up to ~600 bp in length, which is generally adequate for classification to the genus level. Several open-source bioinformatic pipelines, including QIIME [76] and mothur [77], are freely available, which will cluster the rDNA reads into operational taxonomic units (OTUs), assign taxonomy and perform various diversity analyses. It is important to consider several technical factors that could lead to systematic biases in the final community composition, including the choice of universal primers, as mentioned above, as well as the DNA extraction method, which should extract DNA equally from all members of the population [78,79].

A drawback of rDNA-based phylogenetic analysis is that, due to the relatively high conservation of this gene, its resolution is limited and cannot be used to distinguish between different strains. A wide range of other molecular typing techniques is available for strain-level typing, which typically infer sequence variability based on DNA fragment length polymorphisms. These methods generate electrophoretic DNA banding patterns from genomic DNA either through restriction enzyme digestion, (e.g., restriction fragment polymorphism (RFLP), pulsed field gel electrophoresis (PFGE)), PCR amplification (e.g., rapid amplification of polymorphic DNA (RAPD), repetitive sequencing-based PCR (REP-PCR), Multiple-locus variable number tandem repeat analysis (MLVA), Denaturing gradient gel electrophoresis (DGGE)) or a combination of both (e.g., amplified fragment length polymorphism (AFLP)) [80]. Additionally, strain-level sequence-based typing methods, such as multi-locus sequence typing (MLST), use several conserved marker genes to phylogenetically classify members of the same species; however, for these methods, a complete genome sequence is first required to develop the assay. The most definitive phylogenetic resolution, of course, is achieved by whole genome sequencing of the isolate, which is now becoming routine due to the reduced cost made possible by NGS.

Although phylogenetic analysis is a useful tool for the identification of functional strains, traditional morphological and biochemical characterization is still necessary to confirm the physiological characteristics of the isolates. These may include light [18,54], phase contrast, bright field [59] and electron microscopy [12,16,57] to determine cell size, shape and Gram-stain type. Biochemical tests, such as carbon source utilization, enzyme activity and chemical sensitivities [18,19,27,33,38,45,46,50,58,59,81], as well as commercial identification systems, such as BIOLOG or API^®^ strips, may also be used for the identification of MBMs [12,21,35,60]. In addition, fatty acid and ribosomal protein profiles may be determined using gas chromatographic analysis of fatty acid methyl esters (GC-FAME) and matrix assisted laser desorption/ionization-time of flight (MALDI-TOF) techniques, respectively [12,59].

A safety assessment must be performed to exclude potential pathogens, which may produce extracellular toxins that could contaminate food and feed. *Bacillus* spp. with mycotoxin biotransformation activities have been more sought after, due to their perceived probiotic properties. However, some species, such as *Bacillus cereus*, are known toxin producers, and even species outside the *Bacillus cereus* group have been reported to produce related enterotoxins [82]. Yi et al. (2011) [21], using two commercial immunoassay kits, evaluated the enterotoxin-producing ability of *Bacillus licheniformis* CK1 by assaying for the Hbl and Nhe enterotoxins, which are produced by *Bacillus cereus*.

It is recommended to check the functional strains against the U.S. Food and Drug Administration (FDA) Generally Recognized as Safe (GRAS) list and European Food Safety Authority (EFSA) Qualified Presumption of Safety (QPS) list. Biological agents belonging to GRAS are exempt from premarket review and FDA approval. Similarly, biological agents included in the QPS list usually undergo a simplified assessment by the EFSA [83,84].

The identified MBMs are suggested to be registered and deposited in a microorganism collection organization for further research or patent procedures. Those governmental or private non-profit organizations include the Agriculture Research Service Culture Collection (NRRL), the International Depositary Authority of Canada (IDAC), Deutsche Sammlung von Mikroorganismen und Zellkulturen (DSMZ) and the American Type Culture Collection (ATCC).

### 3.5. Physiological Characterization of Mycotoxin Biotransformation Activity

In this stage, the isolated microorganisms should be investigated to verify the biotransformation activity by determining the efficiency correlation with cell growth, influencing factors and the position of active substrates. A time course showing a decrease in mycotoxin levels combined with an increase in biotransformation products provides clear evidence of the biotransformation activity of the isolated strain [12,14,30,37,53,61,85,86,87]. The efficiency of biotransformation can be measured based on the relationship between mycotoxin decrease and cell growth. The mycotoxin biotransformation rate (Equation (1)) may be calculated as follows [15,32]:

(1) $$\mathrm{Mycotoxin}\;\mathrm{Biotransformation}\;\mathrm{rate}=\;\frac{\mathrm{amount}\;\mathrm{of}\;\mathrm{transformation}\;\left(\mu g\right)}{\mathrm{amount}\;\mathrm{of}\;\mathrm{dry}\;\mathrm{cell}\;\left(\mathrm{mg}\right)\times \mathrm{time}\;\left(\mathrm{hour}\right)}\;$$

Knowledge of intrinsic and extrinsic factors that influence the biotransformation is beneficial to understanding the mechanisms of enzymatic biotransformation and for developing and optimizing the detoxification conditions in the complex food/feed matrix. A number of studies have evaluated intrinsic factors, including carbon source [26,34], nitrogen source [26,34,88], vitamins [26], metals ions [12,34,46,89,90,91,92,93], enzyme inhibitors and promoters [52,89,90,91,92,93,94], initial concentration of mycotoxins [93,95], initial concentration of cells [53,95] and initial pH value [12,14,17,26,31,34,41,46,54,60,88,89,95,96], as well as extrinsic factors, including temperature [12,14,17,25,26,31,34,38,41,46,52,54,60,88,89,95,96], aeriation (shaking rate) [26], oxygen preference [14], as well as pre-incubation [26] and incubation time [34,97]. The experimental designs and optimized biotransformation conditions are summarized in Table 2. In addition to the investigation of single factors, researchers have also looked for optimized conditions for multiple variables using mathematic models. One example is the optimization of AFB_1_ biotransformation based on six parameters (temperature, pH, liquid volume, inoculum size, agitation speed and incubation time) using a Plackett–Burman design followed by a response surface methodology (RSM) based on a central composite design (CCD). The degradation efficiency reached 95.8% based on the predicted parameters from the mathematic model [97]. Other methods, such as the orthogonal method and one-factor-at-a-time method, were also useful for screening of optimized biotransformation conditions [26,34].

As an observed decrease in the amount of mycotoxin may result from absorption rather than biotransformation, identification of the daughter compound(s) is necessary to confirm biotransformation activities. The most direct evidence to support biotransformation is the observation of new product(s) in parallel with a decrease in the amount of the original mycotoxin. In some cases, however, the biotransformation product is not detectable through chemical analysis methods, and indirect methods must therefore be adopted to distinguish between adsorption and biotransformation. The involvement of an enzymatic reaction, rather than simple binding, is evidenced by the loss of biotransformation activity following heat or acid-inactivation of cells or in an extracellular extract treated with enzyme inactivating agents, such as proteinase K, SDS or EDTA [12,15,19,98,99]. Furthermore, the subcellular location of active components responsible for biotransformation can be identified by comparing activities in the whole cell culture, extracellular extract (by centrifugation) and cytoplasmic extract (by cell lysis) [25,27,34,45,50,59,88,99].

### 3.6. Identification of Mycotoxins and Their Biotransformation Products

To determine the mycotoxin content in a sample, chromatographic techniques are most commonly adopted. Based on the properties of the sample matrices and the mycotoxin itself, a solvent can be chosen for extraction followed by a range of different clean-up procedures. For example, DON was extracted from mouldy corn with 84% acetonitrile (acetonitrile/water 84:16, *v*/*v*) on a rocking platform at 60 RPM for two hours at room temperature [100] before HPLC analysis. ZEA was usually recovered by a mixture of water and methanol (50:50) [18,41] before HPLC analysis. Solvent extraction can be performed under regular shaking conditions or more efficiently with the aid of an ultrasonicator [18]. Table 3 summarizes the solvents used during the extraction of various mycotoxins and can be used as a baseline to choose appropriate extraction solvents for the analysis of mycotoxins.

The strategy for the identification of unknown mycotoxin biotransformation products will be very different from the determination of known mycotoxins in a sample due to the unknown properties of the products. Thus, the sample needs to be subjected to both polar and non-polar solvent extraction and the formation of new compounds identified by comparing the chromatographs of samples before and after biotransformation. Initial identification of the biotransformation products is usually performed using LC/MS, while final identification requires the peaks to be purified at milligram levels and subjected to NMR analysis. For example, the DON biotransformation product 3-*epi*-DON by *D. mutans* 17-2-E-8 was purified from bacterial culture following centrifugation, filtration, freeze-drying and high-speed countercurrent chromatography. The purified 3-*epi*-DON was then subjected to 1D and 2D NMR, and the structure was then identified [102]. Metabolomic strategies have started gaining recognition for the identification of new products resulting from microbial biotransformation. With the progress in metabolomics and its related tools and software, the identification of novel biotransformation mycotoxin products will become easier and faster.

### 3.7. Evaluation of Toxicity of Biotransforming Product

While many studies report novel structural/chemical modifications that render mycotoxins less toxic [116,117,118,119], the final use of any biological detoxification method is dependent on providing empirical evidence for this claim. Furthermore, some country/state regulatory agencies have set up rigid parameters for the assessment of the safety and efficacy of any developed detoxification product, which need to be addressed accordingly before the registration of that product. A very clear example is the EU regulations regarding the additives for use in animal nutrition and the establishment of a new functional group of feed additives (No. 1831/2003 and No. 386/2009).

Different in vitro and in vivo [120,121,122] approaches were suggested in the past to test for changes in toxicity (Table 4), each with a unique set of advantages and challenges [123,124]. The adopted system should take into consideration: (a) the nature of the tested mycotoxin as screening carcinogens for example with long-term exposure studies is different from screening estrogenic toxins; (b) host suitability and selection since mono-gastric animals for example show higher sensitivity towards certain toxins (such as DON) in comparison to other livestock; (c) the solubility and stability of final biotransformation products (the reduced solubility and stability of 3-*keto*-DON for example necessitates using organic solvents for storing and diluting this metabolite that might interfere with assay outcomes in certain cases.); and finally, (d) the route of exposure; even though the oral route is the main contamination means, symptoms might also develop in animals due to inhalation of grain dust (as the case of aflatoxins for example) [125,126]. Generally speaking, a host highly ranked within the evolutionary tree is more sensitive toward mycotoxins; hence, bacterial hosts are the least preferred for evaluating the bio-toxicity of mycotoxins.

The high costs of raising and keeping large animals and strict guidelines for using animals in research [132,133] are other factors to consider when performing preliminary toxicity evaluations. In some cases, the use of primary and secondary cell lines derived from different hosts (mainly porcine/canine) [134,135,136,137,138] may be warranted to replace actual animals. Specific cell lines may be more or less suitable for testing certain toxins. For example, He et al. (2015) [127] used heterogeneous human epithelial colorectal adenocarcinoma (Caco-2) cells and mouse embryonic fibroblast (3T3) cells to assess cell viability and DNA synthesis, respectively, and thereby to illustrate the diminished toxicity of 3-*epi*-DON. Recently, mouse models have been gaining more attention and becoming feasible routes for bio-potency testing. He et al. (2015) [127] followed the above cell culture assays by exposing B6C3F1 mice to DON and 3-*epi*-DON treatments for 14 consecutive days, demonstrating that toxin-induced lesions were only observed in the adrenal glands, thymus, stomach, spleen and colon of the DON-group, but not 3-*epi*-DON. Some alternative approaches for using large animals in toxicity testing have been devised recently. For example, explants of specific and more sensitive tissues were developed by the Oswald group using pig jejunal explants to investigate the toxicity of deepoxy-DON and 3-*epi*-DON [139,140].

Another primary challenge for toxicity testing is the purification of enough starting materials/metabolites with which to proceed. The optimum range to start with can span milligram to gram quantities, based on the selected host, assay and testing approach. In some cases, the development of a large-scale preparative purification of the final biotransformation metabolite(s) is unavoidable in order to proceed with the toxicity testing. He et al. (2015) [102] applied a refined high-speed counter-current chromatography protocol to scale up the purification of 3-*epi*-DON from *D. mutans* 17-2-E-8 bacterial cultures before toxicity assessment.

The stability of the final biotransformation products used in toxicity bio-assays is another factor that should be considered. Conjugation-based modifications (acetylation, glycosylation, phosphorylation) are usually more difficult to address as the attached groups can be hydrolyzed or cleaved under the implemented experimental conditions. Compounds with chemically-active groups, such as ketones [141,142] (3-*keto*-deoxynivalenol for example), are another example of unstable modifications that can give misleading results, as such reactive groups seek the simultaneous reduction to more thermodynamically-stable alcohols (DON or 3-*epi*-DON) in aqueous solutions with the possibility of reverting back to the original toxins (DON in the above example).

Beyond showing a reduction in the toxicity of the final biotransformation product(s), the mechanism of this reduction must also be determined [143,144]. For example, the reduction in DON toxicity due to C3 carbon epimerization was attributed to attenuated binding of 3-*epi*-DON within the ribosomal peptidyl transferase center [139,140], in addition to increased polarity of *3*-*epi*-DON and decreased molecular interactions with different cellular targets, such as *Fusarium graminearum* Tri101 acetyltransferase [145]. Similar results were also obtained for deepoxy-DON when transformed anaerobically by strain BBSH 797 [139,140]. This mechanistic information is pivotal for predicting how the final biotransformation product(s) will behave under different scenarios and usage conditions, such as under unfavorable pH conditions, or in the presence of metabolizing bacteria in the animal intestinal tract. Some of the modifications that were promising in early stages (conjugation-based modifications such as acetylation/glycosylation) were subsequently found to be incompatible with practical agricultural and industrial applications as they give rise to so-called masked mycotoxins [146,147], where the modifying chemical groups are easily cleaved or hydrolyzed by intestinal bacteria [148,149,150,151,152].

### 3.8. Genes and Enzymes

One of the ultimate goals for mycotoxin mitigation strategies is to establish the conditions needed for efficient practical usage [9]. In many agricultural and industrial applications, the use of whole bacterial cells, for instance through incorporation into fermentation matrixes (especially if they belong to the lactic acid group), might be the most feasible approach to mitigation [153,154]. This also reduces the costs associated with enzyme(s) purification and the need to incorporate any co-factors/co-substrates into the final enzymatic reactions. The above scenario is not always possible due to either processing conditions that do not favor the use of microorganisms (human food chain applications) or the presence of natural consortium that outcompete the active strain(s). In such cases, the use of pure enzyme preparations is unavoidable.

The identification of functional genes and the purification of detoxification enzyme(s) is a laborious and time-consuming process that involves working expertise in the fields of microbiology, analytical and synthetic chemistry, molecular biology, enzymology, genomics and bioinformatics. For detailed information regarding such a process and the involved procedures, the readers are referred to other recently published in-depth reviews [9,155,156,157].

After the responsible enzyme(s) are identified, a second stage of troubleshooting and optimization is initiated to deliver workable enzymatic mixtures that are suitable for the chosen application. Many native enzymes are not stable under certain processing conditions (pH, temperature, presence of inhibitors) or require specific expensive co-factors, such as NADP(H), to function. Such properties can sometimes be manipulated to make these enzymes more industry friendly through targeted protein-engineering [158,159]. The enantioselective epoxidation of styrene by two-component monooxygenases offers a good example of efficient enzyme engineering that was preformed recently. A fusion protein was generated by joining the C-terminus of *StyA* epoxidase with the N-terminus of the *StyB* reductase. Furthermore, the above fusion protein was cloned into a single-vector expression system to couple the epoxidation function to NADH oxidation, thereby enhancing the overall catalytic function of the system. The observed positive changes in the catalytic mechanism were attributed in part to an increased flavin-binding affinity of the *StyB* reductase associated with its N-terminal extension [160]. In a similar optimization, the NADP(H) dependence of hyperthermophilic 6-phosphogluconate dehydrogenase was engineered to favor the less expensive NAD(H) cofactor for a promising industrial application in bio-batteries [161].

### 3.9. Feasibility of Commercial Application

From the application point of view, there are many additional challenges to overcome in order to achieve success. These obstacles include the validation of detoxification activities in food/feed materials, the industrial-scale production and marketing authorization based on the extensive assessment. Unlike ideal in vitro conditions, many factors such as poor nutrient availability, lower pH, natural temperature, interactions with the microbiota and complicated structures of food/feed materials may become hurdles for successful application. For example, the ensiling procedure for production of silage, an important fodder for ruminants, generates an anaerobic environment with acidic pH, high carbon/nitrogen ratio and low nutrient availability, which inhibits most MBMs that grow well and present detoxification activities in media [162]. During the large-scale production of MBMs, the difficulties to maintain the activity may appear as the media and fermentation conditions vary from those in the laboratory. From an economical point of view, the media used in industrial-scale fermentation processes are usually produced from less expensive and more readily available materials, as opposed to the costly synthetic or semi-synthetic media used in laboratory-scale fermentation. Moreover, the downstream processing steps, such as freeze-drying, spray-drying or preparing a direct-fed microbial (DFM) product, may also influence the detoxification activities. Thus, the optimization and validation of mycotoxin detoxification activities should eventually be performed under conditions of commercial-scale production. In addition, any negative or uncertain effects, such as nutrient/palatability loss and safety of biotransformation agents, must also be considered. Finally, the manufacturer should obtain marketing authority before they may claim their products as anti-mycotoxin additives. In Europe, for example, the dossier of anti-mycotoxin additives registration must include the mycotoxin specificity, the species specificity, the efficacy and the safety. The marketing authority in EU member states is issued by the EC based on positive evaluation by the EFSA [163]. For these reasons, the successful commercial application of microbial detoxification has been limited. To our knowledge, Biomin^®^ BBSH 797 is the sole microorganism product that has obtained market authority in the EU. The product has the capacity of biotransforming DON to harmless products and was approved as a pig feed additive in 2013. Recently, the EFSA published a positive scientific opinion on the safety and efficacy of Biomin^®^ BBSH 797 for application in poultry feed. FUMzyme^®^, a purified enzyme isolated from the fumonisin-degrading soil bacteria *Sphingopyxis* sp. MTA 144, is the only enzyme product for mycotoxin detoxification that has been approved by the EC [164,165,166].

The feasibility of commercial applications has been evaluated in a number of research papers and granted patents [8,9]. One of the essential considerations at this stage is to validate the detoxification activity of MBMs in the complex food/feed matrices to which they will be applied, such as corn, wheat, barley, DGGS, peanut meal, pistachio nut, rice straw, apple juice, apple puree and grape must [13,20,32,37,38,39,47,53,54,100,108,110,167]. The typical procedure includes preparation of samples with either spiked or naturally-contaminated mycotoxins, incubation of samples with the MBMs under natural conditions, followed by extraction and chemical analysis of mycotoxins and their metabolites. Ito et al. (2012) verified a decrease in DON in 1000 kernels of wheat and barley grain by applying *Marmoricola* sp. MIM116. The authors also investigated spreading agents and selected 0.01% Tween 80 for its advantages in facilitating cell growth and DON reduction [32]. *Gluconobacter oxydans*, a bacterium isolated from PAT-contaminated apples, has been reported to degrade 96% of PAT (800 µg/mL) to ascladiols in apple juice. However, a full evaluation of the quality attributes of apple juice, the toxicity of ascladiol and safety assessment of the MBM is needed before any industrial applications of such a microorganism [37].

Since the principle detrimental effect of mycotoxin contamination is the decreased growth of livestock, another consideration for the application of MBMs is the effect of detoxified feed on livestock performance. A two by two factorial design is recommended for these studies. Specifically, the experimental samples should be catalogued into four groups including positive mycotoxin-free control, negative mycotoxin-exposed control, mycotoxin-free with detoxifying agents and mycotoxin-exposed with detoxifying agents. The strength of this design is that it not only verifies the detoxification activity, but also ensures that the bio-availability of essential feed ingredients is not impaired by the detoxifying agent [168]. Li et al. (2011) [100] designed an animal trial to evaluate the growth performance of swine, a DON-sensitive livestock, fed a diet detoxified by the isolate *Bacillus* sp. LS100. Compared to pigs fed a diet with *Fusarium*-infected corn with DON, the daily feed consumption, daily weight gain and feed efficiency of pigs fed the LS100-detoxified diet were significantly improved by 45%, 82% and 32%, respectively. These results demonstrate that microbially-detoxified feed can be used in the livestock industry to reduce the effects of DON toxicity, while not conferring significant negative effects on the nutrition and palatability of the feed.

In addition to reducing mycotoxin contamination through biotransformation, some detoxification strategies have introduced specific microorganisms with additional benefits to commercial applications. For example, *Bacillus subtilis* ANSB01G may be delivered as spores, which have the ability to tolerate the gut environment. In addition, this strain produces antimicrobial compounds against common bacterial pathogens such as *Escherichia coli*, *Salmonella typhimurium* and *Staphylococcus aureus*, which could improve the growth of livestock [19]. In another study, a *Bacillus licheniformis* strain was shown to have higher xylanase, CMCase and protease activities, which may enhance the digestibility of nutrients in feed [21]. Another potential advantage of detoxifying microorganisms is that certain such microorganisms may possess antifungal activity, inhibiting the growth of mycotoxin-producing fungi and further reducing mycotoxin contaminations [33,35,47,95].

## 4. Conclusions and Research Trends

The biological detoxification of mycotoxins is an attractive and environmentally-friendly alternative to the chemical and physical decontamination methods explored extensively over the past three decades [169]. The recently-reported examples, coupled with the emergence of some efficient commercialized biological/enzymatic agents, highlight the promise of this approach to address the safety of animal feed and human food [170,171].

The introduction of state-of-the-art research tools, such as next-generation sequencing, recombinant-enzyme overexpression and robust HPLC-MS/MS systems, combined with our enhanced understanding of the actual mechanisms underlying the diminished toxicity of final biotransformation products [140,146,172], will immensely aid in the identification [12], optimization and usage [170,171] of such naturally-derived alternatives. It is anticipated that these novel approaches will form the basis for sustainable long-term solutions to the ever-growing problem of mycotoxins in the coming years.

## Figures and Tables

**Table 1:** Strategies and methodologies in the enrichment and isolation of mycotoxin biotransforming microorganisms. DGGE, denaturing gradient gel electrophoresis; T-RFLP, terminal restriction fragment length polymorphism.

Mycotoxin	Enrichment		Isolation			
	Medium	Strategy/Methodology	Medium	Strategy/Methodology	Biotransforming Strains	Reference
DON	Corn meal broth	Soil samples enriched from the corn contaminated by the DON-producing fungi	Corn meal agar	Single colonies screening; extended incubation time for slow-growth strains	*Devosia mutans* 17-2-E-8	[12]
	Anaerobic incubation medium with 10% chicken cecal digesta extract	In vivo enrichment with moldy wheat; antibiotics treatment; guiding the enrichment by PCR-DGGE	L10 agar	Single colonies screening	*Bacillus* sp. LS-100	[43]
	M10 medium + DON (100 µg/mL)	Treatment by antibiotics and hemin	M1 medium	Single colonies screening	*Eubacterium* sp. BBSH 797	[55,56]
	Mineral salts with peptone medium + DON (50 µg/mL)	Antibiotics and heat treatment; guiding the enrichment by T-RFLP	Mineral salts with peptone agar	Single colonies screening; extended incubation time for slow-growth strains	Microbial consortium with at least 6 bacterial genera	[14]
	Mineral medium + DON (100 µg/mL)	DON as a sole carbon source	1/100 nutrient agar	Single colonies screening	*Nocardioides* sp. WSN05-2	[13]
	Mineral salt medium + DON (100 µg/mL)	In situ plant enrichment in contaminated wheat head by spraying DON	MRDG medium	Single colonies screening; using gellan gum rather than agar	*Marmoricola* sp. MIM116	[32]
	Mineral medium + DON (100 µg/mL)	DON as a sole carbon source	Reasoner’s 2A (R2A) agar, 1/100 nutrient agar	Single colonies screening	9 *Nocardioides* spp. and 4 *Devosia* spp.	[15]
	BYE medium + DON (200 µg/mL)	Repeated sub-culturing in fresh medium with high level of DON (200 µg/mL)	1/10 nutrient agar	Single colonies screening	E3-39 (belonging to *Agrobacterium* or *Rhizobium*)	[16]
	Inorganic salt culture medium + DON (4 µg/mL)	Enrichment with minimal nutrients	Czapek’s agar, LB agar	Single colonies screening	*Aspergillus tubingensis* NJA-1	[57]
	Mineral salts with peptone medium + DON (50 µg/mL)	In situ soil enrichment by spraying DON; guiding the enrichment by PCR-DGGE	-	-	Microbial consortium	[17]
ZEA	Minimal salt medium + ZEA (2 µg/mL)	ZEA as a sole carbon source	LB agar	Single colonies screening	*Pseudomonas alcaliphila* TH-C1, *Pseudomonas plecoglossicida* TH-L1	[18]
	LB broth	Selective screening *Bacillus* strains by heat treatment	LB agar	Single colonies screening	*Bacillus subtilis* ANSB01G	[19]
	Minimal salt medium + ZEA (2 µg/mL)	ZEA as a sole carbon source	LB agar	Single colonies screening	*Pseudomonas otitidis* TH-N1	[41]
	M1 + ZEA (25 µg/mL) + nystatin (15 µg/mL), M2 + ZEA (500 µg/mL)	ZEA as a sole carbon source	Nutrient agar	Single colonies screening	Acinetobacter sp. SM04	[23]
	M9 medium + ZEA (50 µg/mL)	ZEA as a sole carbon source	LB agar	Single colonies screening	Microbial consortium	[24]
AFB_1_	Coumarin medium (with 1% coumarin)	Coumarin, a basic molecular structure of aflatoxins, as a sole carbon source	Coumarin medium	Single colonies screening; coumarin as a sole carbon source	*Stenotrophomonas maltophilia* 35-3	[46]
	Coumarin medium (with 1% coumarin)	Coumarin, a basic molecular structure of aflatoxins, as a sole carbon source	Coumarin medium	Single colonies screening; coumarin as a sole carbon source	*Pseudomonas aeruginosa* N17-1	[25]
	-	-	Modified Hormisch medium (with 0.1% coumarin)	Coumarin as a sole carbon source	*Stenotrophomonas* sp. NMO-3	[58]
	Nutrient broth	Non-selective enrichment	Coumarin medium (with 0.1% coumarin)	Single colonies screening; coumarin as a sole carbon source; K-B disk diffusion	*Aspergillus niger* ND-1	[34]
	Minimal salt/vitamin medium + fluoranthene (10 mg/mL)	Fluoranthene as a sole carbon source	R2A agar	Single colonies screening	*Mycobacterium fluoranthenivorans* sp. nov.	[59]
	Minimal salt medium + AFB_1_ (10 µg/mL)	AFB_1_ as a sole carbon source	Minimal salt agar + AFB_1_ (10 µg/mL)	Single colonies screening	*Bacillus* sp. TUBF1	[31]
	-	-	Nutrient agar	Single colonies screening	*Bacillus licheniformis* CM21, *Bacillus subtilis* MHS 13	[35]
AFB_1_, AFM_1_, AFG_1_	LB broth	Selective screening *Bacillus* strains by heat treatment	LB agar	Single colonies screening	*Bacillus subtilis* ANSB060	[45]
PAT	Mineral salt medium + increased concentration (300–600 µg/mL) of PAT	PAT as a sole carbon source	Mineral salt agar + PAT (600 µg/mL)	Single colonies screening	*Byssochlamys nivea* FF1-2	[54]
	-	-	YEPD medium + PAT (10 µg/mL)	Screening in liquid medium	*Kodameae ohmeri* HYJM34	[60]
CIT	Mineral broth + (1–4 µg/mL) of CIT	CIT as a sole carbon source	Mineral salt agar + CIT (10 µg/mL)	Single colonies screening	*Klebsiella pneumoniae* NPUST-B11	[26]
	Mineral broth + CIT (1 µg/mL)	CIT as a sole carbon source	Mineral salt agar + CIT (1–5 µg/mL)	Single colonies screening	*Rhizobium borbori* PS45	[27]
	-	-	Nutrient agar	Screening strains by disc plate diffusion assay (50 µg/disk of CIT)	*Moraxella* sp. MB1	[50]
FUB_1_	BYE medium + FUB_1_ (500 µg/mL)	Increasing population of FUB_1_-transforming microbes; antibiotics treatment	Nutrient agar (NA), NA + sucrose, NA + skim milk, PYEI agar, BYE agar	Single colonies screening	NCB 1492 (belonging to *Delftia* or *Comamonas*)	[28]
OTA	-	-	YES medium + OTA (2 µg/mL)	Screening in liquid medium	*Aspergillus niger* CBS 120.49	[61]
	-	-	Czapek-Dox medium + OTA (40 µg/plate)	Screening point-pated colonies by observing the loss of fluorescence	*Acinetobacter calcoaceticus NRRL* B-551	[62]
	-	-	LB agar + OTA (3 µg/mL); medium with isocoumarin as the sole carbon source	Screening microbes using isocoumarin as a sole carbon source	*Bacillus subtilis* CW 14	[47]

**Table 2:** Intrinsic and extrinsic factors to influence the biotransformation rates.

Factor	Mycotoxin	Biotransforming Product(s)	Optimal Condition/Reverse Effect
Carbon source	AFB_1_	U.I. ^a^	Starch (4.0%) [34]
	CIT	U.I.	Glucose (1.2%) [26]
Nitrogen source	AFB_1_	U.I.	Yeast extract (0.5%) [88]; tryptone (0.5%) [34]
	CIT	U.I.	Peptone (0.3%) [26]
Vitamins	CIT	U.I.	Vitamin C (100 µg/mL) [26]
Metals ions	DON	3-*epi*-DON	Minerals added in the corn steep liquor and peptone [12]
	ZEA	U.I.	Zn^2+^, Mn^2+^, Ca^2+^, Mg^2+^ (10 mmol/L) [89]
	AFB_1_	U.I.	Mg^2+^, Cu^2+^ (10 mmol/L) [46]; Ca^2+^, Mg^2+^ (10 mmol/L) [90,91,92]; Mn^2+^, Cu^2+^ (10 mmol/L) [25]; Mg^2+^, Zn^2+^, Cu^2+^, Mn^2+^ (10 mmol/L) [93]
Enzyme inhibitor/enhancer	ZEA	U.I.	Reverse effect: chelating agents of EDTA, OPT (10 mmol/L) [89]
	AFB_1_	U.I.	Reverse effect: chelating agents of EDTA, OPT (10 mmol/L) [90,91,92]
	OTA	OTα	Reverse effect: chelating agents of EDTA (10 mmol/L), OPT (1 mmol/L) [52]
	AFB_1_	U.I.	Tween 80, Triton X-100 (0.05%) [93]
	AFB_1_	U.I.	NADPH (0.2 mmol/L), NaIO_4_ (3 mmol/L) [94]
Concentration of mycotoxins	AFB_1_	U.I.	0.5 µg/mL [93]
	OTA	U.I. ^a^	0.1 µg/mL [95]
Concentration of cells	OTA	U.I.	10^8^ CFU/mL [95]
	OTA	OTα	10^9^ CFU/mL [53]
Initial pH	DON	3-*epi*-DON	pH = 7 [12]
	DON	DOM-1	pH = 6.5–7 [14]; pH = 5–10 [17]
	ZEA	U.I.	pH = 7–8 [89]; pH = 4.5 [41]
	AFB_1_	U.I.	pH = 5–6 [96]; pH = 6–7 [31,34,88]; pH = 8 [46]
	PAT	*E*- and *Z*-ascladiol	pH = 3–6 [60]
	PAT	U.I.	pH = 3–5 [54]
	CIT	U.I.	pH = 7 [26]
	OTA	U.I.	pH = 4 [95]
Temperature	DON	3-*epi*-DON	20–35 °C [12]
	DON	DOM-1	20–35 °C [14]; 20–37 °C [17]
	ZEA	U.I.	30–37 °C [41]; 42 °C [89]
	AFB_1_	U.I.	30–37 °C [31,34,46,88,96]
	PAT	*E*- and *Z*-ascladiol	35 °C [60]
	PAT	U.I.	37 °C [54]
	CIT	U.I.	37 °C [26]
	OTA	OTα	25–35 °C [52]
	OTA	U.I.	28 °C [95]
Shaking rate	CIT	U.I.	200 RPM [26]
Oxygen preference	DON	DOM-1	Aerobic condition [14]
Concentration of mycotoxins	OTA	U.I.	0.1 µg/mL [95]
Pre-incubation time	CIT	U.I.	36–48 h [26]

^a^ U.I. means unidentified.

**Table 3:** Analytical methods for the detection and identification of mycotoxins and their biotransforming products.

Mycotoxins/Biotransforming Products	Extraction Solvents	Analytical Method
DON	50% methanol [12,43,48]; 84% acetonitrile [100,101]; ethyl acetate [30]	HPLC [12,13,15,100,101]; LC-MS [48,101,102]; ELISA [32]; HSCCC [101]; NMR [102]
3-*epi*-DON	50% methanol [12]; ethyl acetate [13]	HPLC [12,13,15]; LC-MS [102]; NMR [13,102]
DOM-1 (deepoxy DON)	84% acetonitrile [100]; 50% methanol [48]	HPLC [100]; LC-MS [14,48]; GC-MS [17]; MS [43]
3-*keto*-DON	Ethyl acetate [16,30]	MS [16,30]; NMR [16,30]
ZEA	50% methanol [18,23,41]; 90% acetonitrile [19]; 84% acetonitrile [21]; 50% acetonitrile [98]	HPLC [18,19,21,24,41]; LC-MS [23,89,98]; ELISA [24]
1-(3,5-dihydroxy-phenyl)-10′-hydroxy-1′*E*-undecene-6′-one	Chloroform [22]	TLC, MS, NMR [22]
ZEA-sulfate	60% methanol [36]	LC-MS [36]
ZEA-4-*O*-β-glucoside		TLC, MS, NMR, IR [103]
α-ZAL, β-ZAL, α-ZOL, β-ZOL, ZAN, 8′(*S*)-hydroxyzearalenone, ZEA-2,4-bis(methyl ether), ZEA-2-methyl ether	50% chloroform [104]	TLC, MS, NMR, IR [104]
ZEN-4-*O*-sulfate	33% chloroform:methanol (9:1) [105]	MS, NMR, infrared [105]
ZOM-1 ((5*S*)-5-({2,4-dihydroxy-6-[(1*E*)-5-hydroxypent-1-en-1-yl]benzoyl}oxy)hexanoic acid)	Ethyl acetate [106]	HPLC, LC-MS, NMR [106]
α-ZOL, α-ZOL-S, ZEA-14-sulfate, ZEA-16-sulfate, ZEA-14-Glc, ZEA-16-Glc	50% acetonitrile [107]	LC-MS [107]
AFB_1_	60% methanol [108]; 50% methanol [45]; chloroform [38,46,88,109]; dichloromethane [110]	HPLC [25,38,45,46,88,96,108,109,110]; TLC [38,96,109]; ESMS [109]; LC-MS [38,96,109]; HR-FTMS [96]; ELISA [34]
AFB_2_	Chloroform [25]	HPLC [25]
AFG_1_	60% methanol [108]; 50% methanol [45]	HPLC [45,108]
AFM_1_	Chloroform [25]; 50% methanol [45]	HPLC [25,45]
AFD_1_, AFD_2_, AFD_3_	Chloroform [111]	TLC, HPLC, GC-MS, FT-IR [111]
PAT	Ethyl acetate [54,60,99]	TLC [85]; HPLC([37,54,60,85]; LC-MS [99]; NMR [85]
DPA (desoxypatulinic acid)	Ethyl acetate [99]	HPLC [112]; LC-MS [99]; NMR [112]
*E*- and *Z*-ascladiol	Ethyl acetate [60,85]	TLC [85]; HPLC [37,60,85]; LC-MS [37,60]; NMR [37,85]
CIT	Acetone:ethyl acetate (1:1) [27]; ethyl acetate [50]	TLC(C04); HPLC [26,27]
Decarboxycitrinin	Ethyl acetate [50]	MS, NMR [50]
FUB_1_		TLC [28]; HPLC [28]; GC-MS[28]; LC-MS [113,114]
Heptadecanone, isononadecene, octadecenal, eicosane		GC-MS [28]
Hydrolyzed FUB_1_		LC-MS [113,114]
2-keto-hydrolyzed FUB_1_		LC-MS, NMR [115]
OTA	Methanol [51]; dichloromethane [33,52,61]; ethyl acetate [53]	TLC [52,61]; HPLC [33,51,52,53,61]
OTα	Methanol [51]; dichloromethane [52];	HPLC[51,52]; LC-MS [51]
l-β-phenylalanine	Methanol [51]	HPLC [51]

**Table 4:** Models and methodologies of toxicity evaluation of mycotoxins and their biotransforming products.

Mycotoxins	Biotransforming Products	Model	Methodologies	Reference
DON	3-*epi*-DON	Caco-2 cells	Evaluation of metabolic activity by an MTT cell proliferation assay	[127]
	3-*epi*-DON	3T3 cells	Evaluation of DNA synthesis activity by a cell proliferation ELISA employing BrdU incorporation	[127]
	3-*epi*-DON	Female B6C3F_1_ mice	Evaluation of effects on body weight gain, relative organ weights, food consumption, hematology and clinical chemistry	[127]
	DOM-1	Swine kidney cells	Evaluation of metabolic activity by an MTT cell proliferation assay	[44]
	DOM-1	Chicken lymphocytes	Evaluation of DNA synthesis activity by a cell proliferation ELISA employing BrdU incorporation	[56]
	DOM-1	Starter pigs	Evaluation of effects on growth performance and serum metabolites	[100]
	3-*keto*-DON	Mouse spleen lymphocytes	Evaluation of immunosuppressive activity by a cell proliferation assay	[16]
ZEA	1-(3,5-dihydroxy-phenyl)-10′-hydroxy-1′*E*-undecene-6′-one	MCF-7 cells	Evaluation of estrogenic activity by a WST cell proliferation assay	[22]
	ZEA-sulfate	MCF-7 cells	Evaluation of estrogenic activity by an MTS cell proliferation assay	[36]
	α-ZAL, β-ZAL, α-ZOL, β-ZOL	MCF-7 and MDA-MB-231 cells	Evaluation of estrogenic activity by an MTT cell proliferation assay	[128]
	α-ZAL, β-ZAL, α-ZOL, β-ZOL, ZAN, 8′(*S*)-Hydroxyzearalenone, ZEA-2,4-bis(methyl ether), ZEA-2-methyl ether	Rat uteri	Evaluation of relative binding affinity by an estrogen receptor binding assay	[104]
	ZOM-1	Yeast YZRM7	Evaluation of estrogenic activity by a sensitive yeast assay	[106]
	ZOM-1	Human estrogen receptor-α	Evaluation of estrogenic activity by a HitHunter EFC estrogen chemiluminescence assay	[106]
	U.I. ^a^	Pre-pubertal female gilts	Evaluation of effects on growth performance, genital organs, serum hormones and histopathological changes	[129]
	U.I.	Yeast BLYES	Evaluation of estrogenic activity by a sensitive yeast assay	[130]
	U.I.	Pre-pubertal female rats	Evaluation of estrogenic activity by an immature uterotrophic assay	[130]
AFB_1_	AFD_1_, AFD_2_, AFD_3_	Hela cells	Evaluation of cytotoxicity by an MTT cell proliferation assay	[111]
	U.I.	L929 cells	Evaluation of cytotoxicity by an MTT cell proliferation assay	[108]
	U.I.	*Salmonella typhimurium* TA100	Evaluation of mutagenicity by an Ames assay	[109]
	U.I.	*Artemia salina*	Evaluation of toxicity by an insect larvae survival assay	[31]
PAT	DPA	*Escherichia coli*	Evaluation of microbial toxicity	[99]
	DPA	Seeds of *Arabidopsis thaliana*	Evaluation of phytotoxicity	[99]
	DPA	Human hepatocytes LO2	Evaluation of cytotoxicity by an MTT cell proliferation assay	[99]
	DPA	Human lymphocytes	Evaluation of cytotoxicity by a trypan blue cell proliferation assay	[112]
CIT	U.I.	*Bacillus subtilis* TISTR	Evaluation of microbial toxicity	[27]
OTA	U.I.	HepG2 cells	Evaluation of cytotoxicity by an MTT cell proliferation assay	[95]
	OTα	Zebrafish (*Danio rerio*) embryo	Evaluation of teratogenicity	[131]

^a^ U.I. means unidentified.

## Abbreviations

AFB_1_	aflatoxin B_1_
AFB_2_	aflatoxin B_2_
AFG_1_	aflatoxin G_1_
AFM_1_	aflatoxin M_1_
CIT	citrinin
DON	deoxynivalenol
DOM-1	deepoxy-deoxynivalenol
DPA	desoxypatulinic acid
FUM	fumonisin
FUB_1_	fumonisin B_1_
NIV	nivalenol
OTA	ochratoxin A
OTα	ochratoxin α
PAT	patulin
ZEA	zearalenone
ZAL	zearalanol
ZOL	zearalenol
ZAN	zearalenone
ZOM-1	((5*S*)-5-({2,4-dihydroxy-6-[(1*E*)-5-hydroxypent-1-en-1-yl]benzoyl}oxy)hexanoic acid)
